# WORKING WITH NATIVE HAWAIIANS TO RECLAIM TRADITIONAL PRACTICES

**DOI:** 10.1093/geroni/igae098.1729

**Published:** 2024-12-31

**Authors:** Kathryn Braun, Noreen Mokuau, Kukuna Yoshimoto

**Affiliations:** University of Hawaii, Honolulu, Hawaii, United States; University of Hawaii, Honolulu, Hawaii, United States; University of Hawaii, Honolulu, Hawaii, United States

## Abstract

With colonization, Native Hawaiians were strongly discouraged from speaking Hawaiian and practicing their traditions, and these practices went underground. Thus, few elders today know the language or traditional Hawaiian practices. Amendments to the Hawai‘i Constitution in 1978 supported the bringing back of language and culture. For example, Native Hawaiian immersions schools began to be funded by the state, and today students can earn their elementary, secondary, and tertiary degrees in the Hawaiian language. However, after years of being hidden, much of the knowledge of language and traditional practices had to be resurrected and, to some extent, recreated. The 3-person research team, which included two Native Hawaiian scholars, collected stories from Native Hawaiian kumu loea (expert teachers) of traditional practices, soliciting information on how they learned their craft, how they are currently practicing it, and how they are teaching others. Interviewees included practitioners of Hawaiian spirituality, care of the dead, land management, healthy eating, language, song, conflict resolution, herbal healing, traditional massage, cordage, weapons, martial arts, law, and medicine. Analysis included several rounds of member checking. Findings illustrate how knowledge was passed down despite its suppression and the challenges interviewees faced accruing knowledge as adult learners, rather than as native oral learners. All demonstrated personal resilience, dedication to culture, and pride in their role in bringing back practices that were almost lost. The research process could be replicated by other investigators dedicated to hearing and documenting the process of indigenous knowledge regeneration.

